# Outcome and quality of life in obese patients underwent laparoscopic vs. open appendectomy

**DOI:** 10.1186/s12893-022-01732-7

**Published:** 2022-07-23

**Authors:** Ahmed H. Hussein, Abdullah El-Baaly, Waleed M. Ghareeb, Khaled Madbouly, Haitham Gabr

**Affiliations:** 1 General Surgery Department, Faculty of Medicine, Suez Canal University, Ismailia, Egypt; 2 Colorectal Surgery Unit, Faculty of Medicine, Alexandria University, Alexandria, Egypt; 3 Gastrointestinal Surgery Unit, General Surgery Department, Faculty of Medicine, Suez Canal University, Ismailia, Egypt

**Keywords:** Laparoscopy, Appendicitis, Obesity, Quality of life

## Abstract

**Background:**

Although obesity is a popular reason for choosing laparoscopic appendectomy (LA) versus open appendectomy (OA), however, the question of whether there is a difference remains. Our goal is to investigate if there is a difference between OA and LA in obese patients.

**Methods:**

Fifty-eight obese patients diagnosed with acute appendicitis according to ALVARDO score at department of surgery at Suez Canal university hospitals from March 2020 till August 2021 were included. The study participants were assigned in two groups LA and OA. This study aimed to comparing between LA and OA regarding intraoperative complications, length of hospital stays, post -operative pain, and rate of post-operative complications. Meanwhile, using SF-36 scoring questionnaire, the quality of life was compared between both groups.

**Results:**

A total of 58 patients were included in the present study (LG = 29 patients and OG = 29 patients). The early post-operative complications (within 30 days after surgery) were significantly lower in the LA group (5 patients out of 29) than the OA (11 patients out of 29). Additionally, lower incidence of complications was noticed in the LA group (2 out of 29 patients) compared to OA (6 patients out of 29) beyond 30 days after operation. Patients with laparoscopic surgery had statistically significant higher overall quality of life scores (SF-36) (72 ± 32) compared to open surgery patients (66 ± 35) 2 weeks after operation**.**

**Conclusion:**

The laparoscopic procedure was associated with lower incidence of post operative complications. However**,** open appendectomy was superior for a shorter operative time. Laparoscopic approach is not only used for therapeutic purposes, but also it has a diagnostic role.

## Introduction

The gold-standard treatment for acute appendicitis is to perform an appendectomy. With the development in the field of surgery the quest has been raised to treat various surgical ailments by minimally invasive techniques [1]. With their less invasive nature and positive results, laparoscopic procedures are becoming more popular in gastrointestinal surgeries. The number of open interventions, especially for benign diseases like cholecystectomy and appendectomy, has decreased dramatically [2, 3].

Laparoscopy also outperforms appendectomy in terms of wound site infections, postoperative recovery time, and out-of-hospital expenditures, according to medical research [4, 5]. However, there is a link between laparoscopy and specific scenarios, such as an increased ratio of intra-abdominal abscess and higher hospital charges, according to the research [6]. Because the abdominal wall of obese people is thicker, it may be more challenging to disclose the surgical field, execute surgical methods, and deal with wound-related difficulties. Laparoscopy resolves these difficulties, leading to the opinion that laparoscopic appendicectomy (LA) is superior to open appendectomy (OA) in the treatment of appendicitis. Although some studies suggest that LA is a secure and effective approach for both acute and perforated appendicitis, other data suggests that the open procedure is preferable [3, 7, 8].

With the rising number of people who are obese (body mass index [BMI] > 30), and the number predicted to rise, it is critical to evaluate if LA is beneficial for obese patients with appendicitis. Although obesity is a popular reason for choosing LA versus OA, however, the question of whether there is a difference remains. Our goal is to investigate if there is a difference between OA and LA in patients who are classified by their BMI.

## Patients and methods

### Study design and setting

This is a prospective study on obese patients with acute appendicitis treated with appendicectomy at Suez Canal University Hospitals in the period of March 2020 through December 2021. Ethical approval for the study was obtained from the Institutional Review Board of Suez Canal University, Faculty of Medicine (code: #4245/2020). The study was registered at the www.clinicaltrial.gov under number: NCT05434988 at 28/06/2022.

### Selection criteria and outcome measurement

**Fig. 1:**
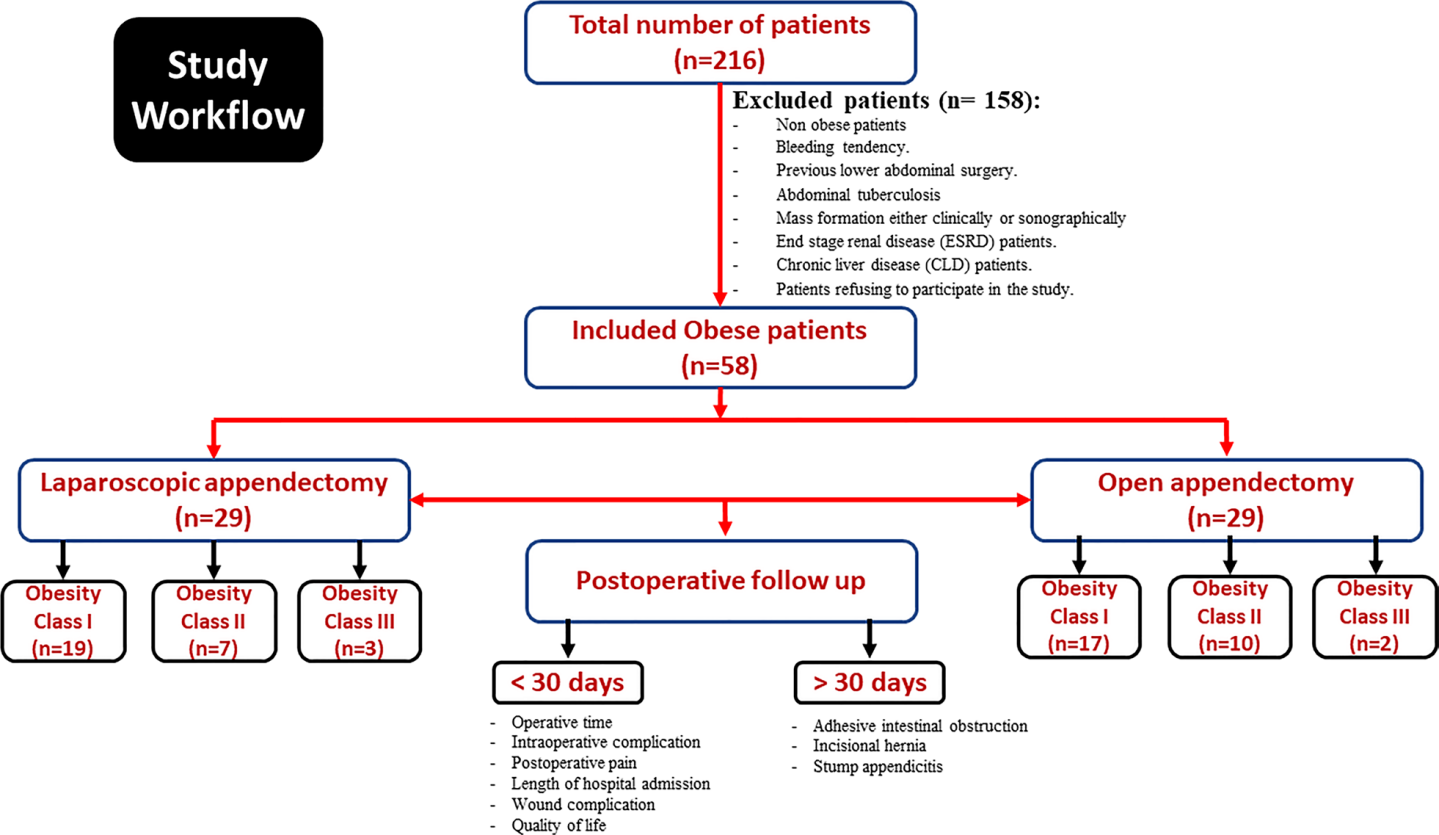
Flow chart depicting the inclusion and exclusion approached for the current study participants

Obese patients were divided into laparoscopic group (LG) and open group (OG). Patients aged between 18 and 40 years, with BMI ≥ 30 kg/m^2^ and diagnosed as acute appendicitis according to ALVARDO score i.e., history of right lower quadrant pain or peri-umbilical pain shifting to the right lower quadrant with nausea and/or vomiting, fever of more than 38 °C, right lower quadrant guarding, and tenderness on physical examination and/or leukocytosis above 10,000 cells per ml were included. In this is non- randomized study, the included patients were enrolled in each group on a consecutive basis at range of 1 (for open approach): 1 (for laparoscopic approach). We excluded patients bleeding tendency, previous lower abdominal surgery, abdominal tuberculosis, mass formation either clinically or by ultrasound, end stage renal disease (ESRD) patients, and patients refusing to participate in the study (Fig. 1).

The main outcome of the present study was to compare the LG vs. OG in terms of intraoperative and postoperative complications during the 30 days postoperative. Furthermore, the quality of life has been compared between both groups using SF-36 scoring questionnaire [9].

### Port placement for laparoscopic appendicectomy

Different 3 surgical teams were contributed to this study. Laparoscopic appendectomy procedure is typically performed under general anesthesia. There are several port placements in laparoscopic appendectomy. The main principle is triangulation of instrument ports to visualize and expose the appendix clearly. The first trocar (10 mm) for the optical device is introduced peri-umbilically, followed by two 5 mm trocars, one in the right lower quadrant just above the pubis (to grasp the appendix) and the other in the left iliac fossa (for the (right-handed) surgeon's right hand), assuming the appendix is in its normal anatomic position. The locations of the 5 mm trocars can be changed based on the anatomic position of the appendix as determined before to surgery (for example a subhepatic appendix could lead to placing the trocars as for cholecystectomy) [10].

### Sample size and statistical analysis

The sample size was determined using an online sample size calculator (http://www.raosoft.com/samplesize.html). The proportion of postoperative complication among obese patients with laparoscopic appendectomy and open appendectomy was considered 6.6% and 9.1%, respectively [11]. Assuming that the population size (patients presenting with suspected acute appendicitis during the study period) is 350 patients of those 105 (30%) are obese (according to the incidence of obesity in Egypt), a minimum sample size of 58 patients was required, with the margin of error set at 5% and confidence level of 95%. P-values of less than 0.05 were used to denote statistical significance at a 95% level of confidence.

Categorical variables were expressed as frequencies and percentages, while continuous variables were expressed as mean ± standard deviation (SD). Categorical variables were compared using Chi-Square or Fisher's exact test. Continuous variables were compared using Student t-tests or Mann–Whitney test, as appropriate. All statistical analyses were performed using IBM SPSS software (Version 25.0. Armonk, NY: IBM Corp.). A p-value < 0.05 was considered significant.

## Results

### Characteristics of patients

A total of 58 patients were included in the present study (LG = 29 patients and OG = 29 patients). The mean age of patients underwent laparoscopic appendectomy was 48.9 years while patients operated through open approach were 48.1 (P = 0.96). More than 60% of the patients in both groups were females while hypertension was the most common chronic illness among the studied patients (Table 1).

**Table 1:** Comparison between laparoscopic and open groups in regard to baseline characteristics of the patients

Variables	Lap group (n = 29)	Open group (n = 29)	p-value
Age (years), mean ± SD	48.95 ± 8.49	48.12 ± 11.06	0.96
Gender, n (%)			
Male	12 (40)	10 (34.5)	0.29
Female	17 (60)	19 (65.5)	
BMI			
Obese class I (30–34.9 kg/m^2^)	19 (65.5)	17 (58.6)	0.76
Obese class II (35–39.9 kg/m^2^)	7 (24.1)	10(34.4)	
Obese class III (Morbidly obese) (≥ 40 kg/m^2^)	3 (10.4)	2 (6.8)	
Chronic illness, n (%)			
Absent	19 (65.6)	20 (69)	0.54
Present	10 (34.4)	9 (31)	
Hypertension	6 (20.6)	7 (24.1)	
Diabetes	8 (27.5)	4 (13.7)	
IHD	0 (0)	2 (6.8)	
Symptoms			
Rt. iliac fossa pain	27 (93.1)	29 (100)	0.957
Anorexia	25 (86.2)	23 (79.3)	0.220
Nausea and vomiting	10 (34.4)	14 (48.2)	0.140
Fever	19 (65.5)	23 (79.3)	0.091
Rebound tenderness	28 (96.5)	29 (100)	1.00
Hemoglobin (gm/ dl)	12.32 ± 1.12	11.86 ± 3.31	0.16
TLC	12.42 ± 4.87	11.39 ± 1.28	0.26
The degree of appendicitis			
Normal (negative appendectomy)	3 (10.4%)	2 (6.8%)	
Non-complicated Acute appendicitis	22 (75.9%)	23 (79.3%)	
Complicated (total)	4 (13.7%)	4 (13.7%)	
Perforated	2	1	
Gangrenous	2	3	

BMI: Body Mass Index

Statistical significance at P < 0.05

### Comparing the intra and postoperative outcome between LG vs. OG

The patients in laparoscopic group had significantly higher operative time (74.32 ± 1.12) compared to patients in open surgery (47.86 ± 3.31) (p < 0.001). Meanwhile, for postoperative pain assessment, the patients in LG had significantly lower visual analogue score (VAS) (3.4 ± 0.56) compared to patients in OG (5.7 ± 1.36) (p < 0.001). Furthermore, LG had significantly lower length of stay in hospital (1.6 ± 0.2) compared to OG (2.4 ± 0.2) (p = 0.011). With following up patients for 30 days postoperative, overall, there was no difference between both groups regarding the development of pelvic abscess, postoperative ileus, and intestinal fistula. However, there was significant difference between both groups in terms of all types of wound complications; wound infection (p 0.04), seroma formation (p 0.001), and wound dehiscence (p 0.02). LG patients had statistically significant higher overall quality of life score (72 ± 32) compared to OG (66 ± 35) 2 weeks after operation (Table 2).

**Table 2:** Comparison between lap and open groups in regard to complications with follow up in < 30 days

Variables	Lap group (n = 29)	Open group (n = 29)	p-value
Operative time (min)	74.32 ± 1.12	47.86 ± 3.31	< 0.001*
Post-operative pain (VAS)	3.4 ± 0.56	5.7 ± 1.36	< 0.001*
length of hospital stay (days)	1.6 ± 0.2	2.4 ± 0.2	0.011*
Bleeding	1 (3.4)	1 (3.4)	
Absent	24 (82.7)	18 (62.1)	0.077
Present	5 (17.2)	11 (37.9)	
Clavien-Dindo classification			
Grade I	1	2	
Grade II	3	7	
Grade III	1	2	
Grade IV	0	0	
Pelvic abscess	2 (6.8)	3 (10.3)	
Paralytic ileus	1 (3.4)	5 (17.2)	
Intestinal fistula	1 (3.4)	2 (6.8)	
Wound complication			
Wound infection	3 (10.3)	10 (34.4)	0.043*
Wound seroma	4 (13.7)	13 (44.8)	< 0.001*
Wound dehiscence	1 (3.4)	5 (217.24.1)	0.022*
Quality of life (SF-36) after 2 weeks	72 ± 32	66 ± 35	< 0.001*

^*^Statistical significance at P < 0.05

After 30 days postoperative follow up, although OG had higher late post-operative complications rate (20.6%) compared to LG (6.8%). However, there was no statistical significance (p = 0.069) (Table 3).

**Table 3:** Comparison between lap and open groups in regard to late post-operative complications (> 30 days)

Variables	Lap group (n = 29)	Open group (n = 29)	p-value
Absent	27 (93.1)	23 (79.3)	0.069
Present	2(6.8)	6 (20.6)	
Adhesive intestinal obstruction	1 (3.4)	4 (13.7)	
Incisional hernia	0 (0)	2 (6.8)	
Stump appendicitis	1 (3.4)	0 (0)	

^*^Statistical significance at P < 0.05

### Discussion

Less post-operative pain, a rapid return to everyday life and activities, and aesthetic benefits have all made laparoscopic treatments more popular than open surgical techniques. Previous research on appendectomy has shown that laparoscopic appendectomy (LA) is preferable to open appendectomy (OA) because of the lower risk of intra-operative complications, fever surgical site infections, and shorter hospital stays in obese patients. Because the abdominal wall of obese people is thicker, it may be more difficult to disclose the surgical field, execute surgical methods, and deal with wound-related difficulties. Laparoscopy resolves these difficulties, leading to the opinion that laparoscopy is superior than open appendectomy (OA) in the treatment of appendicitis [12].

Unlike laparoscopic cholecystectomy for cholecystolithiasis, laparoscopic appendicitis surgery has yet becoming the mainstay of treatment. Given the increased difficulty of abdominal surgery in patients with morbid obesity, selecting the best appendectomy approach for this patient population becomes even more critical [13].

In the present study we included 58 obese patients diagnosed with acute appendicitis from March 2020 till August 2021. The study participants were assigned in two groups: laparoscopic appendectomy group and open appendectomy group. There was no significant difference between the 2 groups in terms of age (p = 0.96), sex (p = 0.29), BMI value (p = 0.76). In addition, there was no significant difference in comorbidities between the 2 groups (p = 0.54). This matching is required to decrease factors that may affect the comparison between the two approaches.

Akbulut et al., suggested that a relationship exists between demographic features, histopathological findings of appendectomy specimens, seasons, days of the week, and working days in patients undergoing appendectomy [14]. Meanwhile the current study, focused on the surgical approach, fund that patients in the laparoscopic group had significantly higher operative time (74.32 ± 1.12) compared to patients in the open surgery group (47.86 ± 3.31) (p < 0.001). In studies on patients with obesity, there is contradictory information regarding the duration of the operation. For example, in a randomized prospective study by Clarke et al., no difference was found between durations in patients undergoing LA and OA [12]. A meta-analysis dated 2004 where 54 studies were analyzed; LA was shown to have a correlation between it and higher cost with an increased risk of prolonged operation time [15]. In contrast, Özozan et al. reported more operative time in minutes in OA group (61 (40–119)) in comparison to the LA group (45 (29–134)) p = 0.002 [16]. Similarly Katar et al. found longer operative duration in OA than in LA (p =  < 0.001) [13]. A meta-analysis in 2015 showed significantly shorter operating times in the laparoscopic appendectomy group compared to open group with a mean difference of -13.96 (-15.44 to − 12.49; P < 0.00001) [1]. These results are also supported by the findings of Mason et al. and Corneille et al. [17, 18]. The varying results may be due to the unequal experiences of the teams studying obese patients.

Attempting to reduce the negative appendectomy and perforation rates, Akbulut et al. have investigated preoperative factors which are shown to be promising to accurately predict the degree of appendicitis [19]. We assume that the presenting study can play a complementary role to Akbulut’s approach. Thus, when suspecting patients to have either negative or perforated appendicitis, laparoscopic approach would be very valuable to explore the abdomen through the single incision of the camera port first. Accordingly, the surgeon can decide further steps that should be leveraged depending on the findings.

Furthermore, Akbulut et al. have highlighted a precious study regarding the unusual histopathological findings in appendectomy specimens. They have reported the existence of some rare cases during routine pathological examination such as cases of enterobiasis, carcinoids, mucinous cystadenomas, eosinophilic infiltrations, mucoceles, tuberculosis, goblet-cell carcinoid, and neurogenic hyperplasia [20]. Although the present study did not have such pathological types, we need to highlight that the laparoscopic approach may have a potential usefulness in such cases. However, further investigations are still needed.

Patients in laparoscopic group had significantly lower visual analogue score (VAS) (3.4 ± 0.56) compared to patients in open surgery (5.7 ± 1.36) (p < 0.001) in the current study which came in concordance with the previous studies. In a recent meta-analysis by Sauerland et al. (56 studies comparing LA versus OA), significant decreases were noted in post-operative pain and time to return to work in patients who received LA [21]. In the 2004s meta-analysis, LA was shown to result in distinctly less pain than OA [15].

In the current study, patients who underwent laparoscopic surgery spent considerably less time in the hospital (1.6 0.2) than those who underwent open surgery (2.4 0.2) (p = 0.011). Similarly, Katar et al. discovered that patients who received LA had a shorter hospital stay than those who underwent OA [22, 23]. Obese individuals who have laparoscopic appendectomy had a considerably lower LOS, according to a meta-analysis published in 2015. Overall, there was a 2.03-day difference in LOS between the two groups (95 percent CI 1.86e2.19 days, p = 0.000) [1]. A study of people with severe obesity revealed similar outcomes [24]. Özozan and his colleagues, on the other hand, discovered no statistically significant difference in the length of hospital stay between the groups (p = 0.181). When it comes to discharging a patient, professional discretion can have an impact on how long they stay in the hospital. The severity of the infection detected during the operation is also a factor that influences the length of hospital stay for appendectomy patients. Patients with ruptured or gangrenous appendicitis may require antibiotic medication after surgery, which may lengthen their stay in the hospital. However, the present work is limited by the small sample size and being a single center study.

### Conclusion

The laparoscopic procedure was associated with lower rates of wound complications, less post-operative pain, and shorter hospital stay, less adhesions and intestinal obstruction complication, open appendectomy was superior for a shorter operative time. Patients with laparoscopic surgery had statistically significant higher overall quality of life scores compared to open surgery patients 2 weeks after operation and Laparoscopic procedure is not only used for therapeutic purposes, but also it has a diagnostic role.

## Data Availability

All data are available with the corresponding author on reasonable request.
